# The Effect of Different Rhizobial Symbionts on the Composition and Diversity of Rhizosphere Microorganisms of Chickpea in Different Soils

**DOI:** 10.3390/plants12193421

**Published:** 2023-09-28

**Authors:** Junjie Zhang, Nan Wang, Shuo Li, Jingqi Wang, Yufeng Feng, Entao Wang, Youguo Li, Tao Yang, Wenfeng Chen

**Affiliations:** 1 College of Food and Bioengineering, Zhengzhou University of Light Industry, Zhengzhou 450002, China; 2 Collaborative Innovation Center for Food Production and Safety of Henan Province, Zhengzhou 450002, China; 3 Departamento de Microbiología, Escuela Nacional de Ciencias Biológicas, Instituto Politécnico Nacional, Ciudad de Mexico C.P. 11340, Mexico; 4 State Key Laboratory of Agricultural Microbiology, Huazhong Agricultural University, Wuhan 430070, China; 5 Institute of Crop Sciences, Chinese Academy of Agricultural Sciences, Beijing 100081, China; 6 College of Biological Sciences, Rhizobium Research Center, China Agricultural University, Beijing 100193, China

**Keywords:** *Mesorhizobium ciceri*, *Mesorhizobium muleiense*, Pseudomonas, competitive ability, chickpea

## Abstract

Background: Chickpea (*Cicer arietinum* L.) is currently the third most important legume crop in the world. It could form root nodules with its symbiotic rhizobia in soils and perform bio-nitrogen fixation. *Mesorhizobium ciceri* is a prevalent species in the world, except China, where *Mesorhizobium muleiense* is the main species associated with chickpea. There were significant differences in the competitive ability between *M. ciceri* and *M. muleiense* in sterilized and unsterilized soils collected from Xinjiang, China, where chickpea has been grown long term. In unsterilized soils, *M. muleiense* was more competitive than *M. ciceri*, while in sterilized soils, the opposite was the case. In addition, the competitive ability of *M. ciceri* in soils of new areas of chickpea cultivation was significantly higher than that of *M. muleiense*. It was speculated that there might be some biological factors in Xinjiang soils of China that could differentially affect the competitive nodulation of these two chickpea rhizobia. To address this question, we compared the composition and diversity of microorganisms in the rhizosphere of chickpea inoculated separately with the above two rhizobial species in soils from old and new chickpea-producing regions. Results: Chickpea rhizosphere microbial diversity and composition varied in different areas and were affected significantly due to rhizobial inoculation. In general, eight dominant phyla with 34 dominant genera and 10 dominant phyla with 47 dominant genera were detected in the rhizosphere of chickpea grown in soils of Xinjiang and of the new zones, respectively, with the inoculated rhizobia. Proteobacteria and Actinobacteria were dominant at the phylum level in the rhizosphere of all soils. *Pseudomonas* appeared significantly enriched after inoculation with *M. muleiense* in soils from Xinjiang, a phenomenon not found in the new areas of chickpea cultivation, demonstrating that *Pseudomonas* might be the key biological factor affecting the competitive colonization of *M. muleiense* and *M. ciceri* there. Conclusions: Different chickpea rhizobial inoculations of *M. muleiense* and *M. ciceri* affected the rhizosphere microbial composition in different sampling soils from different chickpea planting areas. Through high throughput sequencing and statistical analysis, it could be found that *Pseudomonas* might be the key microorganism influencing the competitive nodulation of different chickpea rhizobia in different soils, as it is the dominant non-rhizobia community in Xinjiang rhizosphere soils, but not in other areas.

## 1. Introduction

Chickpea (*Cicer arietinum* L.) belongs to the tribe Cicereae, subfamily Papilionaceae of family Fabaceae [1]. Following dry bean and pea, chickpea is the third most important legume in the world, particularly in Asia and the areas surrounding the Mediterranean Sea. It originated in southeastern Turkey dating back 9500 years [2] and has been cultivated in Xinjiang, China for more than 2500 years according to ancient literature [3,4,5]. The planting area of chickpea in China was about 3056 hectares with a total output of 16,368 tons in 2020 (FAO, 2020). Chickpea crops can fix atmospheric nitrogen in symbiosis with rhizobia up to 176 kg N/ha, providing 85% of the nitrogen required by the host legume [4]. Due to its tolerance of drought and low nutrient soils, chickpea has been traditionally cultivated mainly in regions of Xinjiang, Gansu, and Inner Mongolia in China. However, in recent years, it has been introduced and successfully cultivated in Shanxi, Yunnan, and Henan provinces [6].

Nitrogen is an important element which can be derived from dinitrogen (N_2_), which occupies up to 78% of the earth’s atmosphere, via nitrogen fixation. Nitrogen fixation can convert N_2_ into ammonia [7]. Nitrogen fixation is mainly performed in soil by nitrogen-fixing bacteria and archaea [8]. Among nitrogen-fixing bacteria, rhizobia, can not only live in soil as saprophytes but also form root nodules with their host legumes. In these symbiotic root nodules, rhizobia supply nitrogen to their hosts via biological nitrogen fixation and get carbon nutrition feedback from the legume hosts [9,10]. Underlying rhizobia–legume symbiosis is a complex process including rhizobial infection and nodule development, functioning and senescence [11,12]. Although the rhizobia–legumes interaction is well recognized, this mutualistic association is highly specific and widely diverse [13,14]. For instance, *Rhizobium leguminosarum* bv. *trifolii* can infect only clover species (*Trifolium* spp.) [15], whereas *Sinorhizobium fredii* NGR234 exhibits a broad host range and can infect up to 112 legume genera [16]. For a given host, successful infection by rhizobia depends not only on the competitive ability of different rhizobial species but also the ability of rhizobia to cope with various fluctuating environmental factors, including soil properties and soil pH levels [17,18,19,20,21,22].

The yield and quality of chickpea grain are affected by various biotic and abiotic stresses [23], including the effectiveness of N_2_ fixation. Chickpea is a host stringently nodulated by rhizobial species in the genus *Mesorhizobium*, including *M. ciceri* [24], *M. mediterraneum* [25], *M. muleiense* [26], *M. wenxiniae* [27], and some strains belonging to species of *M. loti*, *M. huakuii*, *M. tianshanense*, *M. abyssinicae*, and *M. plurifarium*, which were originally described as microsymbionts of other legumes [28]. Despite their diverse species affiliation, all the chickpea rhizobia harbor similar symbiotic genes (such as *nodC* and *nifH*) [24,25,26,27,28,29,30]. Soils also contain billions of microorganisms, including bacteria and fungi [31]. Rhizobia may compete with these microorganisms in the soil or rhizosphere of their prospective host legume to establish a symbiotic relationship [32]. Leguminous plants, such as *Lotus japonicum*, *Medicago truncatula*, and soybean (*Glycine max*), reportedly play a crucial role in the establishment of bacterial assemblages in the rhizosphere or root, and the symbiosis between rhizobia and legumes is directly affected by the structure of the microbiota in these two compartments [33,34,35,36,37]. Extensive evidence has shown that in China, a large number of indigenous rhizobia exist in soils of the leguminous crop-growing areas, and the indigenous rhizobia may have higher competitive nodulation ability than the foreign rhizobia [38,39]. Our previous studies have revealed that the introduced *M. ciceri* inoculant occupied a lower percentage of chickpea nodules in the traditional planting area of Xinjiang in China than the indigenous *M. muleiense*, but *M. ciceri* presented stronger competitive nodulation ability in soils of the newly cultivated areas [40,41,42]. It was estimated that this difference might be due to the interactions between rhizobia and other soil microorganisms [40].

As the endosymbionts of roots, rhizobia can influence the composition and structure of the rhizosphere microbiota of their host legumes [43], while the surrounding microbes also could affect the survival and activity of rhizobia. Based on the background above, there is a hypothesis that other soil microbes, except rhizobia, may differentially affect the competitive ability of *M. muleiense* and *M. ciceri* to nodulate chickpea. In this study, in order to explore the key biological factors that influence the competitive nodulation of chickpea by *M. muleiense* and *M. ciceri* in different soils, the composition and diversity of bacterial communities in the rhizosphere of chickpea inoculated separately were compared with the two rhizobial species in soils from old and new chickpea-producing regions in China.

## 2. Results

### 2.1. Bacterial Species (OTU) Richness in the Chickpea Rhizosphere with the Different Treatments

The number of merged reads aligned successfully to each sample varied among the treatments, but the coverage indices in all the treatments were ≥98.3% (Table 1 and Figure S3). Among the four soil samples, the numbers of OTUs tended to decrease in the order XR > BR > QR > YR. The lowest OTU numbers were detected in Xinjiang soil across all the inoculation treatments. However, the effects of inoculation treatments on the OTU numbers varied in different soils (Table 1).

**Figure 1:**
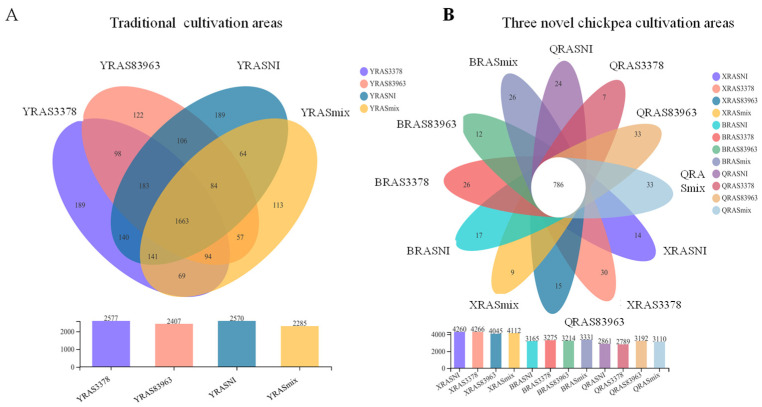
Comparative analysis of 16S rRNA OTUs in soils from traditional cultivation areas of chickpea in Xinjiang and introduced new areas. (**A**) Venn diagram showing unique and overlapped OTUs between different rhizobia inoculation in traditional cultivation area. (**B**) Venn diagram showing unique and overlapped OTUs between different rhizobia inoculation in three novel chickpea cultivation areas.

In Xinjiang soil (Table 1; Figure 1), the blank control (YRASNI) and YRAS83963 (inoculated with *M. muleiense*) had similar numbers of OTUs (2577/2570), which were greater than the OTU numbers in YRAS3378 (inoculated with *M. ciceri*) and YRASmix (inoculated with a mixture of *M. muleiense* and *M. ciceri*) (2407/2285), demonstrating that inoculation with *M. ciceri* apparently decreased bacterial species richness in the chickpea rhizosphere. A total of 3312 OTUs were detected among the four inoculation tests in Xinjiang soil, in which 1663 OTUs were shared by all the inoculation treatments, and 1036 OTUs were shared by two or three treatments. The number of OTUs specific to treatments YRASNI, YRAS3378, YRAS83963, and YRASmix was 189, 189, 122, and 113, respectively. 

In Xinyang (of Henan Province) soil (Figure 1), treatments XRASNI (no inoculation) and XRAS3378 (inoculated with *M. ciceri*) had similar numbers of OTUs (4260/4266), which were greater than those for XRAS83963 (inoculated with *M. ciceri*) and XRASmix (inoculated with a mixture of *M. muleiense* and *M. ciceri*) (4045/4112), demonstrating that inoculation of *M. muleiense* apparently decreased bacterial species richness in the chickpea rhizosphere. A total of 5175 OTUs was detected among the four inoculation treatments in this soil, in which 3055 were common in all the four treatments, while 1577 OTUs were shared by two or three treatments. The treatment-specific OTUs varied from 164 for XRAS3378 to 114 for XRASmix.

In Baicheng (of Jilin Province) soil (Figure 1), the numbers of OTUs increased in the order BRASNI < BRAS83963 < BRAS3378 < BRASmix, indicating that inoculation with both the test strains could increase bacterial species richness in the rhizosphere, but the effect of *M. ciceri* was greater than that of *M. muleiense*. A total of 4087 OTUs was defined in these soil samples with 2374 shared by all four inoculation treatments (Table S1) and 1169 OTUs shared by two or three inoculation treatments. The treatment-specific OTUs varied from 170 for BRASmix to 106 for BRAS83963.

In Qiubei (of Yunnan Province) soil (Figure 1), the numbers of OTUs increased in the order QRAS3378 < QRASNI < QRASmix < QRAS83963, demonstrating that inoculation with *M. muleiense* increased bacterial species richness in the rhizosphere, while the inoculation with *M. ciceri* decreased this value. A total of 4067 OTUs was identified from this soil, with 1997 OTUs shared by all the four inoculation treatments, while 1381 OTUs were shared by two or three inoculation treatments. The treatment specific OTUs varied between 250 (QRASmix) and 128 (QRAS3378).

### 2.2. Bacterial Diversity in the Chickpea Rhizosphere with the Different Treatments

**Figure 2:**
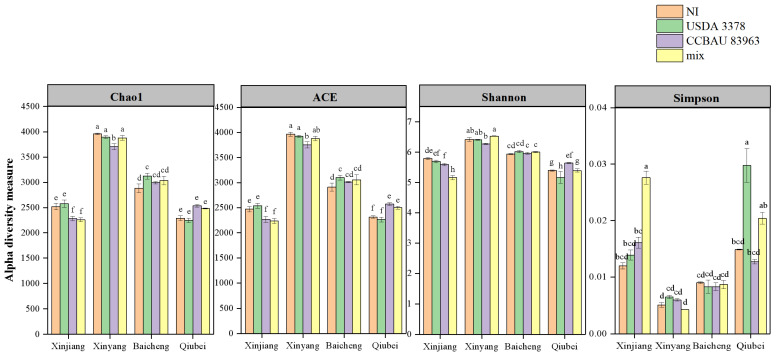
Alpha diversity estimates of the bacterial communities in traditional and novel chickpea culturing area soils. Different lowercase letters between columns indicate significant differences (*p* < 0.05) between corresponding treatments.

The α-diversity values of the rhizosphere bacterial community associated with chickpea grown in the four soil samples calculated on the basis of the 16S rRNA gene sequences are shown in Figure 2. The chao1, ACE, and Shannon indices indicated that the bacterial diversity in the rhizosphere decreased in the order of Xinyang > Baicheng > Xinjiang ≥ Qiubei. This contrasted with the order indicated by the Simpson index of Qiubei ≥ Xinjiang > Baicheng > Xinyang, which suggests that some taxa presented greater dominance in Qiubei and Xinjiang soils than in Baicheng and Xinyang soils.

In Xinjiang soil (Figure 2), the inoculation treatments YRAS83963 and YRASmix significantly decreased the CHAO 1, ACE, and Shannon indices, but increased the Simpson index, compared with the treatment YRASNI. Inoculation with *M. ciceri* USDA 3378^T^ (YRAS3378) did not change the root microbial composition and diversity compared with the blank control (YRASNI), but it produced a coordinative effect with *M. muleiense* CCBAU 83963^T^ in the mixture inoculation treatment (YRASmix). The effects of inoculation in Qiubei soil contrasted with those in Xinjiang soil, e.g., the QRASNI and QRAS3378 treatments gave similar CHAO 1, ACE, and Shannon indices, while these indices were increased with QRAS83963 and QRASmix (except its similar Shannon index with QRASNI). As to the Simpson index, it was increased with QRAS3378, but decreased with QRAS83963, compared with the blank control (QRASNI). In Xinyang soil, the CHAO 1, ACE, and Shannon indices were not significantly different among the rhizosphere microbiomes of XRASNI, XRAS3378, and XRASmix, but these indices were all decreased in treatment XRAS83963, demonstrating that inoculation with *M. muleiense* CCBAU 83963^T^ decreased bacterial diversity in the rhizosphere of chickpea. However, the Simpson index increased in both the single strain inoculation treatments, but significantly decreased in the mixture inoculation, compared with the blank control (XRASNI), indicating that rhizobial mixtures can give different effects to their constituent strains. In Baicheng soil, treatment BRAS3378 increased Chao1 and ACE values, but no significant change was observed in other indices and in the other inoculation treatments, in comparison with the blank control.

### 2.3. Bacterial Community in the Rhizosphere of Chickpea with the Different Treatments

**Figure 3:**
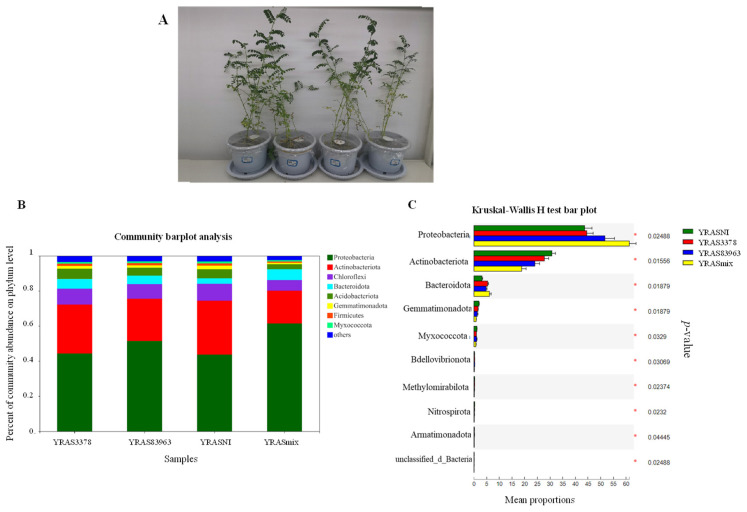
Growth of chickpea and distribution of dominant bacterial phyla in the rhizosphere soil of Xinjiang. (**A**) The growth of chickpea plants planted in the soil of Yinggebao Township, Xinjiang after 45 days; (**B**) horizontal abundance distribution of dominant microbial phyla; (**C**) test analysis of the significance of difference between different rhizosphere soil groups under different treatments in Xinjiang.

The composition of bacterial communities in the four treatments of Xinjiang soil was evaluated for the dominant taxa at different taxonomic levels (Figure 3). A total of eight dominant phyla (with >1% relative abundance) was detected in the four treatments. These phyla were Proteobacteria (50.24%), Actinobacteria (25.36%), Chloroflexi (8.25%), Bacteroidota (4.89%), and Acidobacteriota (4.62%), which accounted for more than 90% of the reads (Figure 3B). Although Proteobacteria and Actinobacteriota were dominant in all the four treatments, accounting for more than 72% of the total reads, the relative abundance of the different phyla varied among the treatments. The Kruskal–Wallis rank sum test (Figure 3C) indicated that rhizobial inoculation significantly changed the bacterial community composition in the chickpea rhizosphere compared with the blank control. For example, the relative abundance increased for Proteobacteria and Bacteroidota, but decreased for Actinobacteriota and Gemmatimonadota. Furthermore, the effects of strain *M. ciceri* USDA 3378^T^ were less than that of *M. muleiense* CCBAU 83963^T^, while these two strains produced an additive effect in the double inoculation treatment (YRASmix).

**Figure 4:**
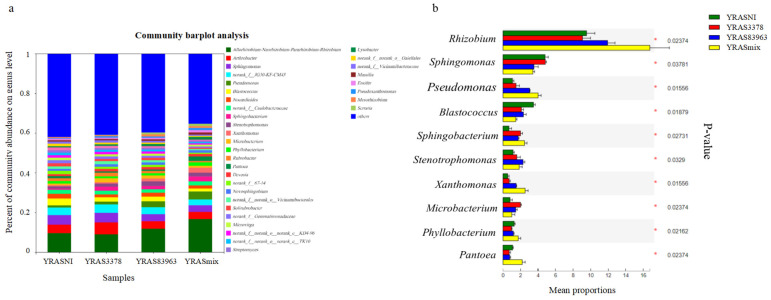
Horizontal abundance distribution of the dominant microbial genera in rhizosphere soil of Xinjiang. Changes in the composition of prokaryotic communities showns in follows: (**a**) among different rhizobial inoculation treatments in three novel chickpea cultation areas. (**b**) Among different rhizobial inoculation treatments in Xinjiang.

Based on OTU annotations, 34 dominant genera (relative abundance > 1%) were detected in the four treatments in Xinjiang soil, accounting for more than 58% of total bacteria in the rhizosphere (Figure 4). The dominant genera were Rhizobium (covering also Allorhizobium-Neorhizobium-Pararhizobium) (11.78%), Arthrobacter (4.37%), Sphingomonas (4.14%), JG30-KF-CM45 (3.60%), Pseudomonas (2.40%), and Blastococcus (2.35%) (Figure 4a). The abundance of the dominant genera in rhizosphere samples varied with the different treatments (*p* < 0.05). In general (Figure 4b), the abundances of Rhizobium, Pseudomonas, Sphingobacterium, Stenotrophomonas, and Xanthomonas were increased, while those of Sphingomonas and Blastococcus were decreased in the YRSA83963 and YRSAmix treatments (*p* < 0.05). The inoculation of *M. ciceri* USDA 3378^T^ (treatment YRAS3378) had no significant effects on abundances of Rhizobium, Pseudomonas, Sphingobacterium, Stenotrophomonas, and Xanthomonas but decreased the abundances of Blastococcus, Phyllobacterium, and Pantoea and increased the abundances of Sphingobacterium and Microbacterium. In addition, the double inoculation further increased the effects of the single inoculations on Rhizobium, Pseudomonas, Sphingobacterium, Blastococcus, and Xanthomonas. The response of Microbacterium and Pantoea to inoculation was interesting because the single inoculation of both strains increased the abundance of Microbacterium and decreased that of Pantoea, but their mixtures had no effects on Microbacterium and increased the abundance of Pantoea, compared with the blank control.

**Figure 5:**
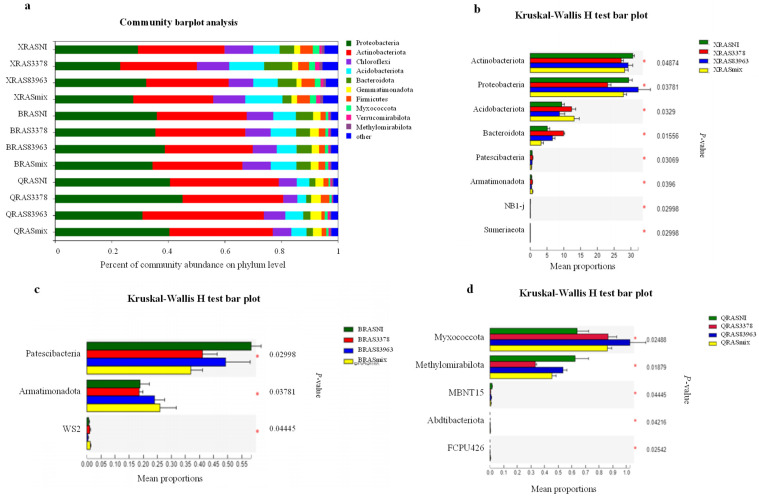
Horizontal abundance distribution of the dominant phyla in rhizosphere soil samples of new areas of chickpea cultivation. Changes in the composition of prokaryotic communities shown: (**a**) among different rhizobial inoculation treatments in three novel chickpea cultation areas, (**b**) among different rhizobial inoculation treatments in Xinyang, (**c**) among different rhizobial inoculation treatments in Baicheng, and (**d**) among different rhizobial inoculation treatments in Qiubei. X, Xinyang, Henan Province; B, Baicheng, Jilin Province; Q, Qiubei, Yunnan Province.

Similarly, the dominant taxa in bacterial communities in the soils from the three novel chickpea culturing regions were evaluated at different taxonomic levels (Figure 5). The dominant phyla detected in the 12 treatments were Proteobacteria (34.51%), Actinobacteriota (32.91%), Chloroflexi (8.66%), Acidobacteriota (8.00%), and Bacteroidota (4.56%), which accounted for more than 85% of the reads (Figure 5a). Proteobacteria and Actinobacteriota were the most common phyla in all the twelve treatments, accounting for more than 60% of the total reads. The relative abundance of Chloroflexi, Acidobacteriota, Bacteroidota, Firmicutes, Myxococcota, Verrucomicrobiota, and Methylomirabilota in Xinyang was greater than that in Qiubei. The relative abundance of Proteobacteria, Actinobacteriota, and Gemmatimonadota in Qiubei was greater than that in Xinyang. The relative abundance of dominant phyla of rhizosphere bacteria in Baicheng was intermediate to that in the other two novel chickpea culturing areas. 

The Kruskal–Wallis rank sum test (Figure 5b) showed that the inoculation treatments XRSA3378 and XRSAmix significantly increased the abundance of Acidobacteriota and Armatimonadota, but decreased the abundance of Actinobacteriota and Proteobacteria, with the greatest increase/decrease in the XRSA3378 treatment. In addition, inoculation of *M. muleiense* CCBAU 83963^T^ (treatment XRAS83963) presented similar effects, but its effects were less on Actinobacteriota, Acidobacteriota, and Bacteroidota, while stronger on Proteobacteria, compared with *M. ciceri* USDA 3378^T^. In Baicheng, all inoculation treatments significantly decreased the abundance of Patescibacteria, and inoculation treatments BRAS83963 and BRASmix increased the abundance of Armatimonadota, with the greatest increase/decrease with the BRSAmix treatment (Figure 5c). Furthermore, the inoculation treatments significantly increased the abundance of Myxococcota, but decreased the abundance of Methylomirabilota and MBNT15 (Figure 5d). Inoculation treatments had the greatest impact on the phylum level abundance of rhizosphere bacteria in Xinyang, followed by Qiubei and Baicheng.

**Figure 6:**
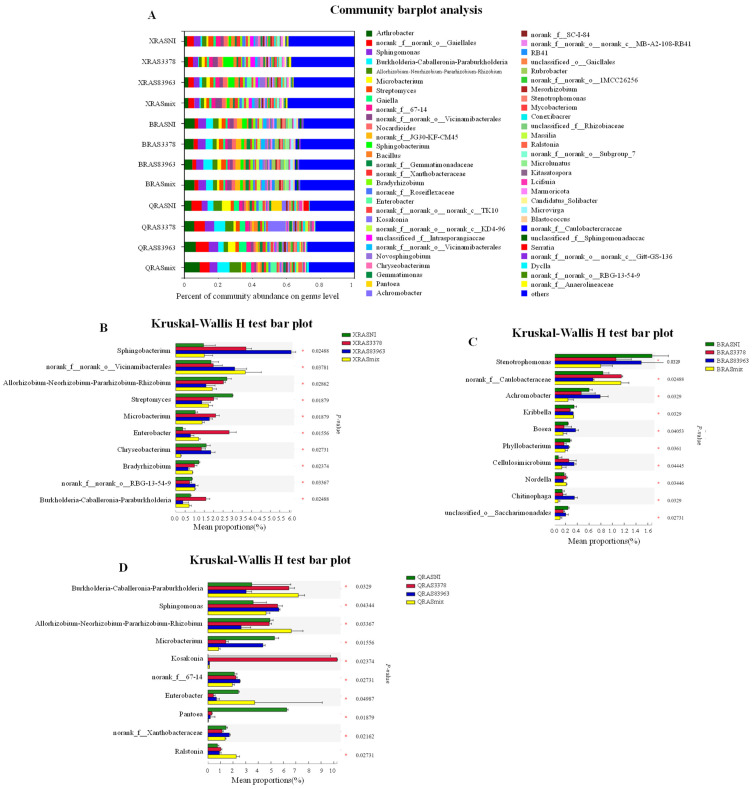
Horizontal abundance distribution of the dominant microbial genera in rhizosphere soil samples from new chickpea-producing regions (**A**). Changes in the composition of prokaryotic communities among different cultivation areas and rhizobial inoculation treatments: (**B**) for Xinyang, (**C**) for Baicheng, and (**D**) for Qiubei.

Based on OTU annotations, 57 dominant genera (relative abundance > 1%) were detected in the twelve treatments in soils of the three novel chickpea culturing areas, accounting for more than 60% of total bacteria in the rhizosphere (Figure 6). The dominant genera were o__Gaiellales, Sphingomonas, Sphingobacterium, o__Vicinamibacterales, Streptomyces, 67–14, f__Intrasporangiaceae, Bacillus, Allorhizobium-Neorhizobium-Pararhizobium-Rhizobium, and Arthrobacter (Figure 6A). In general (Figure 6B), the abundances of o__Vicinamibacterales, Microbacterium, Enterobacter and o__RBG-13-54-9 were increased, while those of Rhizobium (including Allorhizobium-Neorhizobium-Pararhizobium), Streptomyces, Bradyrhizobium, and Burkholderia (including Caballeronia-Paraburkholderia) were decreased in the XRSA83963 and XRSAmix treatments (*p* < 0.05). The inoculation of *M. ciceri* USDA 3378^T^ (treatment YRAS3378) decreased the abundances of Allorhizobium-Neorhizobium-Pararhizobium-Rhizobium, Streptomyces, and Bradyrhizobium but increased the abundances of Sphingobacterium, Microbacterium, Enterobacter, and Burkholderia-Caballeronia-Paraburkholderia. The dominant genera were Arthrobacter, f__Burkholderiaceae, Burkholderia-Caballeronia-Paraburkholderiae, Sphingomonas, JG30-KF-CM45, Streptomyces, and Allorhizobium-Neorhizobium-Pararhizobium-Rhizobium. For the dominant genera (relative abundance of at least 1% in each treatment), only Stenotrophomonas and f__Caulobacteraceae differed significantly among treatments (Figure 6C). The abundance of Stenotrophomonas decreased with the BRAS3378, BRAS83963, and BRASmix treatments, while the abundance of f__Caulobacteraceae increased with BRAS3378 and BRASmix, but decreased with BRAS83963. Distinct inoculation treatments had different effects on the abundance of dominant microorganisms in Qiubei (Figure 6D). The inoculation of *M. ciceri* USDA 3378^T^ (treatment QRAS3378) increased the abundances of Sphingomonas and decreased the abundances of Microbacterium, Pantoea, and f__Xanthobacteraceae. The inoculation of *M. muleiense* CCBAU 83963^T^ (treatment QRAS83963) increased the abundances of Sphingomonas and 67–14, but decreased the abundances of Pantoea and Allorhizobium-Neorhizobium-Pararhizobium-Rhizobium. In addition, the mixture inoculation (QRASmix) increased the abundances of Allorhizobium-Neorhizobium-Pararhizobium-Rhizobium and Ralstonia but decreased the abundances of Microbacterium and Pantoea: Pantoea showed an inverse response to the inoculation of CCBAU 83963^T^. The abundance of Sphingobacterium changed only in XRAS3378 and XRAS83963 but was not affected in other treatments. Sphingobacterium and f__Caulobacteraceae only showed responses to *M. ciceri* USDA 3378^T^ in rhizosphere of Baicheng soil. The abundance of dominant genera in rhizosphere soils of the other novel chickpea culturing areas did not have obvious similarities.

### 2.4. Inoculation Effects on Rhizosphere Microbiota and Exploration of Key Microorganisms 

Based on linear discriminant analysis (LDA), the effect of inoculation on rhizosphere bacteria in Xinjiang was greater than that for the three new chickpea-producing regions (LDA score > 3.0) (Supplementary Figure S2). Allorhizobium-Neorhizobium-Pararhizobium-Rhizobium showed the highest LDA score in Xinjiang, followed by Pseudomonas and Xanthomonas. Sphingobacterium showed the highest LDA score in Xinyang soil, followed by Enterobacter and o_Vicinamibacterales. Stenotrophomonas showed the highest LDA score in Baicheng soil. Kosakonia showed the highest LDA score in Qiubei, followed by Pantoea and Burkholderia-Caballeronia-Paraburkholderia. By comparing the mixed inoculation of *M. ciceri* USDA3378^T^ and *M. muleiense* CCBAU83363^T^ in different ecological areas of chickpea (competing for the soil environment of nodulation), the commonness and characteristics of dominant microorganisms in different areas were found (Table 2). A total of 24 dominant bacterial genera were detected in XRASmix treatments, including Allorhizobium-Neorhizobium-Pararhizobium-Rhizobium, Pseudomonas, Xanthomonas, Pantoea, Sphingobacterium, f__caulobacteraceae and Stenotrophomonas, with abundances of 16.70%, 4.04%, 2.55%, 2.48%, 2.23%, 1.97%, and 1.88% respectively, which were higher than those in the rhizosphere of the new area of chickpea cultivation. The abundance of the above dominant genera in rhizosphere soil of Xinjiang may be related to the competitive difference between *M. ciceri* and *M. muleiense* in the soil of the traditional chickpea cropping area and novel chickpea cropping areas.

**Figure 7:**
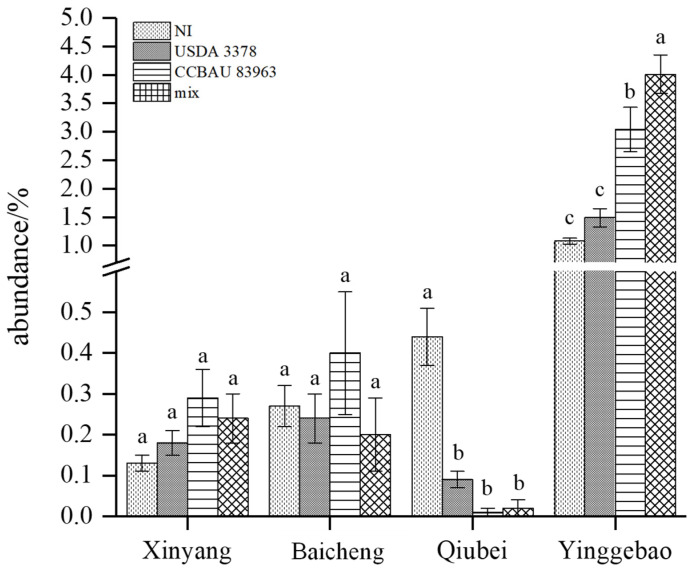
Comparison of *Pseudomonas* abundance in rhizosphere soil of chickpea from different regions. Data are shown as mean ± standard deviation. Different letters indicate significant differences between each column of data (*p* > 0.05).

Among the seven dominant genera, the abundance of Pseudomonas in rhizosphere soil was increased significantly by inoculation with *M. muleiense* CCBAU 83963^T^ and the rhizobial mixture but was unaffected by inoculation with *M. ciceri* USDA 3378^T^. However, the abundance of Pseudomonas did not significantly increase with any of the treatments in soil of new chickpea cultivation areas and significantly decreased in the rhizosphere of Qiubei soil (Figure 7). Xanthomonas abundance showed the same characteristics as Pseudomonas in the traditional chickpea cultivation area but did not occur in the rhizosphere of the new chickpea cultivation areas (Figure 4 and Figure 6). The abundance of Pseudomonas and Xanthomonas in the rhizosphere of Xinjiang soil was not affected by *M. muleiense* CCBAU 83963^T^. In addition, the abundance of Pseudomonas and Xanthomonas in the soil environment was positively correlated with the competitive nodulation response of *M. muleiense*. Therefore, it was preliminarily speculated that Pseudomonas and Xanthomonas might be the key differentiating microorganisms affecting the competitive nodulation ability of *M. ciceri* and *M. muleiense* in the traditional and new chickpea cultivation areas. 

### 2.5. Soil Characteristics and Correlation between Soil Characteristics and Bacterial Composition from Rhizosphere Soils 

The physicochemical characteristics of soil samples are presented in Table 3. The soil samples from the new chickpea cultivation areas had greater OM and TN content than that from Xinjiang. The pH and AP, AK, and TS content of soil in Xinjiang were in the range shown for soils from the new chickpea cultivation areas.

The correlation between rhizobial composition and soil characteristics from different sampling sites (Supplementary Figure S3) showed that *Pseudomonas*, *Xanthomonas*, *Pantoea*, *Rhizobium*, and *Stenotrophomonas* were strongly associated with the site of YGB and their abundance was positively correlated to the pH of the soil. O_Vicinamibacterales were associated with the sites of BC and XY and positive to the TS, AP, TN, and OM of the soil, and *Arthrobacter*, *Sphingomonas* and *Ralstonia* were associated to QB and their abundance was positively correlated with AK of the soil.

## 3. Discussion

Successful symbiosis is jointly regulated by rhizobia and their legume hosts, and the rate of rhizobial nodulation within a given host is variable and influenced by environmental factors and rhizobial symbiont. Legumes have a core rhizosphere microbiota whose composition depends on the genotype of the host [37,44]. Analysis of rhizosphere microbial diversity in traditional chickpea growing areas and new areas showed significant differences in rhizosphere microbial α-diversity among the regions (Figure 1). The highest rhizosphere microbial α-diversity was found in chickpea rhizosphere in Xinyang soil (Henan), which may depend on the original microbial composition of the soil in the different regions. Proteobacteria, Actinobacteriota, Chloroflexi, and Bacteroidetes were the dominant bacterial phyla detected in the rhizosphere soil of chickpea in this study (Figure 2 and Figure 4), which were similar to those in the rhizosphere of soybean [45,46,47], peanut [48,49,50], and bean (*Phaseolus vulgaris*) [51,52,53]. *Rhizobium* (including *Allorhizobium-Neorhizobium-Parararhizobium*) presented high abundance in the rhizosphere of all the studied soils and was one of the core members of the chickpea rhizosphere bacteria (Figure 3 and Figure 5). In addition, *Pseudomonas*, *Arthrobacter*, *Streptomyces*, *Burkholderia*, and some frequently reported phytopathogenic bacteria were widely distributed in the chickpea rhizosphere [54,55,56,57,58,59,60] and showed different abundance variations among inoculation treatments. *Sphingobacterium* and other rarely reported probiotic genera were also present in high abundance in the chickpea rhizosphere, according to Amjad Ali’ s study on chickpea plant growth promoting rhizobacteria in soils in Pakistan [61]. In this study, chickpea rhizosphere soil was collected for analysis after 45 days of crop growth. Previous studies have shown that rhizosphere microorganisms differed significantly between growth periods of legumes, with higher rhizosphere microbial diversity at the middle stage of vegetative growth than at the seedling stage, which may be caused by an increase in the number and diversity of plant rhizosphere exudates [62].

Plant rhizosphere microorganisms are vital for plant health and nutrition. The rhizosphere zone contains a large and diverse community of prokaryotic and eukaryotic microorganisms that can interact with each other and with plant roots. The activity of one member of this community could possibly affect the growth and physiology of other members, as well as the physical and chemical properties of the soil [63]. The different inoculation treatments caused enrichment of different microorganisms in the rhizosphere of chickpea grown in soils of the different ecological zones. Inoculation with exotic rhizobia may affect the composition of the microbial community or the interaction between microbes and the host plant. In this study, we found that the composition of rhizobia in the rhizosphere may also be influenced by other rhizosphere bacteria. *Pseudomonas* may be involved in the colonization of *M. muleiense* and *M. ciceri* in nodules. *Pseudomonas* appeared significantly enriched after inoculation of the indigenous strain of *M. muleiense* in the soil of the traditional cultivation area in Xinjiang, a phenomenon not found in soils of new areas of chickpea introduction. *Pseudomonas* is well known as a beneficial rhizosphere bacterium and is the most dominant non-rhizobial community in the chickpea rhizosphere in Xinjiang soil. Furthermore, *Pseudomonas* has been shown to promote the nodulation and nitrogen fixation capacity of rhizobia [64,65,66,67]. A *Pseudomonas* strain isolated from *Sophora alopecuroides* also promotes plant growth upon reinoculation with *Mesorhizobium* [68]. It was posited that potential microbe–microbe interactions involving *Pseudomonas* also influence the outcome of the root–nodule symbiosis [66]. *Pseudomonas* bacteria have also been shown to colonize root hairs [69] or nodules intercellularly [67]. Interactions among bacteria can be direct, for example filtrates from *Rhizobium* sp. increasing the cell density of *Pseudomonas fluorescens* [70], or mediated via the plant, for example, indoleacetic acid produced by *Pseudomonas* sp. resulted in a more extensive root system in *Galega officinalis* and an increased number of potential infection sites for the compatible *Rhizobium* sp. [71]. Positive interactions have already been seen after the co-inoculation of *Pseudomonas* sp. isolates with a *Mesorhizobium* sp., which led to an increase in nodule number in chickpea [72]. The ability for *Pseudomonas* to selectively colonize healthy plant nodules and reduce the number of ineffective nodules in *L. japonicus* indicated that root–nodule symbiosis is influenced by the broader soil microbiota [66]. In this study, *M. muleiense* CCBAU 83963^T^, the indigenous chickpea rhizobial species in Xinjiang, coexisted in the soil microbiota in the local area together with the other microbial groups, including *Pseudomonas*, to form a harmonious microecology, and these microbes in the soil might improve the nodulation of *M. muleiense* CCBAU 83963^T^, while inhibiting the nodulation of exogenous strain *M. ciceri* USDA 3378^T^. In the newly introduced areas of chickpea, lacking indigenous chickpea rhizobia in the soils, the inoculation of *M. muleiense* CCBAU 83963^T^ and *M. ciceri* USDA 3378^T^ would be more neutral to both of them as the soil microbiota might not improve or inhibit any strain inoculated. In our previous study, it showed that *M. muleiense* CCBAU 83963 was more competitive in Xinjiang, the traditional chickpea plant area, but *M. ciceri* USDA 3378^T^ was a stronger competitor in all the newly introduced areas of chickpea in China [41]. Therefore, this study suggests that *Pseudomonas* may be a key microorganism influencing the competitive nodulation of different chickpea rhizobia populations, and the interactions between *Pseudomonas* and chickpea rhizobia deserve further study. It can be concluded that distinct rhizobial symbionts presented different effects on the composition and diversity of rhizosphere microorganisms.

## 4. Materials and Methods

### 4.1. Experimental Soil and Rhizobia

The soils used in this study were collected from the fields in 2020 after the chickpea crop was harvested, which was August in Xinjiang and Jilin Provinces and June in Henan and Yunnan Provinces, according to the local cropping seasons. From each sampling site, 15 kg of soil was collected at a depth of 0–20 cm with the five-point cross strategy in an area of 10 m × 10 m, and three parallel samples were taken as repeats. The sampling fields were a traditional area of chickpea cultivation in Yinggebao (YR: 45°43′23″ N, 89°57′1″ E) of Mulei County, Xinjiang Autonomous Region, and three new areas of chickpea cultivation, Baicheng City (BR: 45°37′23″ N, 122°48′43″ E) of Jilin Province, Xinyang City (XR: 32°15′36″ N, 114°1′26.4″ E) of Henan Province, and Qiubei County (QR: 23°56′44″ N, 104°18′20″ E) of Yunnan Province (Supplementary Figure S1). Mulei and Baicheng are located in the temperate region, Xinyang is in the subtropical region, and Qiubei is in the tropical region (http://www.diva-gis.org (accessed on 10 December 2021)) of China. Soil samples were transported to the laboratory in sealed boxes for temporary storage at 4 °C. Within one week. a fraction of each repeat soil sample was used for physicochemical analysis of pH, organic matter (OM), total nitrogen (TN), available phosphorus (AP), available potassium (AK), and total salts (TS), as reported previously [73]. Then, the three repeat soil samples from each site were mixed in the same volume to form a final composite sample of the site for the nodulation experiments with the rhizobial strains *M. ciceri* USDA 3378^T^ [6] and/or *M. muleiense* CCBAU 83963^T^ [26].

### 4.2. Experimental Design and Growth Conditions

In this study, *M. ciceri* USDA 3378^T^ and *M. muleiense* CCBAU 83963^T^ were incubated separately in TY broth (tryptone 5.0 g, yeast extract 3.0 g, CaCl_2_ 0.6 g, distilled water 1.0 L, pH 7.2) with shaking at 28 °C for 3–4 days. The cultures were diluted with TY broth to OD_600_ = 0.8 (about 1.7 × 10^9^ CFU/mL) and used as inoculant in the following tests. The experimental treatments were designed as in Table S1. Chickpea (variety Muying 1) seeds were sterilized in 0.2% mercury solution and pre-germinated on 0.8% water-agar medium at 28 °C in the dark. Three germinated seeds with similar root length (about 0.5 cm) were transplanted into each pot containing 0.5 kg of soil sample. The pots with seeds were incubated in an artificial climate incubator for growth with the setting parameters of 16 h of light at 25 °C and 8 h of darkness at 20 °C. Deionized water was added on the first day and then every 4 days according to the needs of the chickpea plants. The humidity was approximately 60%.

### 4.3. Sampling of Rhizosphere Soil

According to the nodulation development observed previously [41], six pots of well-grown chickpea plants were randomly selected from each treatment for collection of rhizosphere soil after 45 days of growth, when the plants were in the middle of the vegetative growth stage. Under aseptic conditions, the aboveground sections of chickpea plants were excised, and the roots were shaken to remove the bulk soil sticking to them; then, the soil attached to the root surface was collected as rhizosphere soil [7,23]. The roots were placed in a centrifuge tube containing 25 mL of sterilized phosphate buffer, and the tube was vortexed at maximum speed for 15 s to release the rhizosphere soil. This process was repeated, and the two turbid solutions were combined. The turbid solution was filtered through a 100 µm sterilized nylon mesh to remove the plant debris and large particles; then, the supernatant was centrifuged at 10,000 rpm for 5 min, and the sediments as rhizosphere soil samples were collected in a 1.5 mL centrifuge tube for storing at −80 °C. Rhizosphere soils were collected in triplicate from each treatment. In total, 48 rhizosphere soil samples (3 repeats, 16 treatments) were used in the subsequent study.

### 4.4. Bacterial 16S rRNA Sequencing

Metagenomic DNA was extracted from 0.5 g of each rhizosphere soil sample using the FastDNA^®^ Spin Kit for Soil (MP Biomedicals, Santa Ana, CA, USA) following the manufacturer’s instructions. The quantity and quality of DNA were measured via electrophoresis in 1.0% (*w*/*v*) agarose gel and a NanoDrop 2000 spectrophotometer, respectively. Using the obtained metagenomic DNA as template, the V3-V4 regions of the bacterial 16S rDNA gene were amplified with the primers 338F (5′-ACTCCTACGGGAGGCAGCA-3′) and 806R (5′-GGACTACHVGGGTWTCTAAT-3′) [74] via PCR as described by Qin et al. [7]. The cycling conditions were as follows: initial denaturation at 95 °C for 3 min, followed by 27 cycles of 30 s at 95 °C, 30 s at 55 °C, 45 s at 72 °C, and finally, at 72 °C for 10 min. The PCR products were detected using agarose gel (2%, *w*/*v*) electrophoresis, recovered from the gel, and purified using an AxyPrep DNA Gel Extraction Kit according to the manufacturer’s instructions. Then, the PCR products were pair-end sequenced (2 × 300 bp) on the Illumina MiSeq platform according to standard protocols at Majorbio Bio-Pharm Technology Co., Ltd. (Shanghai, China). 

### 4.5. Bioinformatics and Statistical Analysis

The fastp (https://github.com/OpenGene/fastp, version 0.20.0 (accessed on 23 August 2023)) software was used to quality control the original sequences acquired in this study. The low-quality sequences were filtered and paired-end reads were assembled using FLASH (http://www.cbcb.umd.edu/software/flash, Version 1.2.7 (accessed on 23 August 2023)) [75]. Subsequently, the clean sequences obtained were clustered with UPARSE into operational taxonomic units (OTUs) at 97% similarity. A representative sequence of each OTU was annotated for species classification using the RDP classifier (http://rdp.cme.msu.edu/, Version 2.2 (accessed on 23 August 2023)) and compared with the Silva 16S rRNA database (V138), using a threshold of 70%. The alpha-diversity indexes of Chao1, Shannon, ACE, Coverage, and Simpson index were calculated via Mothur software (version 1.31.2) [76], and one-way analysis of variance (ANOVA) followed by Duncan’s multiple range test (*p* < 0.05) in R (4.0.3) was used for statistical analysis. Metastats software was used to compare the group abundance of each sample at phylum, class, order, family, and genus levels. Linear discriminant analysis combined with an effect size measurement (LEfSe) was used to find the differentially (*p* < 0.05) abundant taxa across treatments, with an LDA score of at least >3.0 [77].

### 4.6. Correlation between Soil Characteristics and Bacterial Composition from Rhizosphere Soils

Program CANOCO version 4.54 was used to perform the redundancy analysis (RDA) [78] and examine the multiple relationships among the six soil parameters (N, P, K content, OM, TS, and pH), the main bacterial genera from the rhizosphere soils, and the four sampling sites. Before the RDA, a linear or unimodal ordination model was determined using DCA (detrended canonical analysis) [79]. The maximal value of the lengths of the gradient in four ordination axes was below 3, suggesting that the linear gradient analysis model was more suitable, but the unimodal could also be used. The average values (*n* = 3) of soil parameters and the percentage of bacterial genera from different sites were used for the correlation analysis. Permutational multivariate analysis of variance (PERMANOVA) using distance matrices was performed by running the adonis function in the vegan package of R language (R Core Team 2017) with permutations = 999 to determine the influences of the soil factors on the genotype distribution.

## 5. Conclusions

Through high throughput sequencing and statistical analysis, it could be found that rhizobial inoculation of *M. muleiense* and *M. ciceri* affected chickpea rhizosphere microbial composition, which varied in sampling soils from different chickpea planting areas. The abundance of *Pseudomonas* in rhizosphere soil was increased significantly by inoculation with *M. muleiense* CCBAU 83963^T^ and the rhizobial mixture but was unaffected by inoculation with *M. ciceri* USDA 3378^T^ in Xinjiang. However, the abundance of *Pseudomonas* did not significantly increase with any of the treatments in soil of new chickpea cultivation areas. In conclusion, *Pseudomonas* was suggested as a key microorganism influencing the competitive nodulation of different chickpea rhizobia in different soils.

## Figures and Tables

**Table 1:** Quality metrics of pyrosequencing analysis and alpha diversity.

Sample No.	Sample Size	Base NO.	Mean Read	OTU No.	OTUseq
Total	1265838	526583331	415		
YRASNI	51,312.00 ± 1666.87	21251445	414.1700	2577	35930
YRAS3378	49,144.66 ± 1712.33	20366835	414.4113	2407	35332
YRAS83963	45,687.33 ± 3925.27	18951169	414.7993	2570	32341
YRASmix	51,525.33 ± 6203.75	21358247	414.4623	2285	37639
XRASNI	58,066.33 ± 2758.87	24132554	415.613	4260	49386
XRAS3378	60,827.00 ± 435.95	25336443	416.5310	4266	51263
XRAS83963	48,320.00 ± 3928.64	20114175	416.2629	4045	42153
XYRAmix	45,707.33 ± 2526.44	19032936	416.4048	4112	38020
BRASNI	48,381.66 ± 1579.35	20112274	415.7096	3165	42205
BRAS3378	50,884.33 ± 1446.52	21166839	415.9785	3275	43872
BRAS83963	54,192.66 ± 4336.47	22533585	415.8094	3214	46036
BRASmix	55,566.66 ± 5986.33	23098968	415.6967	3331	47569
QRASNI	65,260.66 ± 3544.78	27242438	417.4568	2861	60863
QRAS3378	68,681.66 ± 4046.47	28731629	418.2565	2789	63750
QRAS83963	71,741.66 ± 7174.03	29905753	416.8529	3192	65771
QRASmix	67,219.00 ± 1500.52	28021370	416.8775	3110	62046

**Table 2:** Composition of the most abundant bacterial genera (>1% relative abundance) in the rhizosphere of chickpea grown in different soil samples inoculated with a mixture of *M. ciceri* USDA3378^T^ and *M. muleiense* CCBAU 83963^T^.

Bacterial Genus	Average Relative Abundance (%) in Rhizosphere of Treatment *			
	YRASmix	XRASmix	BRASmix	QRASmix
*Rhizobium*	16.70 ± 1.82	1.9 ± 0.18	2.35 ± 0.25	6.64 ± 0.76
*Pseudomonas*	4.04 ± 0.28	0.24 ± 0.04	0.20 ± 0.07	0.02 ±0.02
*Arthrobacter*	3.52 ± 0.62	2.45 ± 0.17	6.02 ± 0.88	9.19 ± 1.24
*Sphingomonas*	3.41 ± 0.16	3.01 ± 0.27	4.50 ± 0.72	4.62 ± 0.25
JG30-KF-CM45	2.88 ± 0.14	0.72 ± 0.03	3.07 ± 0.14	1.10 ± 0.12
*Xanthomonas*	2.55 ± 0.28	0.00 ± 0.00	0.00 ± 0.00	0.00 ± 0.00
*Sphingobacterium*	2.48 ± 0.23	1.49 ± 0.36	1.48 ± 0.50	0.00 ± 0.00
*Pantoea*	2.23 ± 0.27	0.38 ± 0.03	0.01 ± 0.00	0.02 ± 0.01
f__Caulobacteraceae	1.97 ± 0.07	0.21 ± 0.02	1.13 ± 0.11	0.08 ± 0.01
*Stenotrophomonas*	1.88 ± 0.28	0.63 ± 0.48	0.81 ± 0.16	0.03 ± 0.02
*Nocardioides*	1.61 ± 0.41	2.25 ± 0.08	2.01 ± 0.25	1.08 ± 0.14
*Novosphingobium*	1.21 ± 0.10	2.07 ± 0.32	1.07 ± 0.15	0.12 ± 0.00
*Streptomyces*	0.77 ± 0.02	1.75 ± 0.17	2.41 ± 0.59	2.15 ± 0.18
*Rubrobacter*	0.70 ± 0.97	0.03 ± 0.00	2.20 ± 0.20	0.36 ± 0.05
o__Vicinamibacterales	0.67 ± 0.14	3.72 ± 0.69	2.55 ± 0.26	1.01 ± 0.25
67–14	0.67 ± 0.02	2.42 ± 0.08	1.21 ± 0.15	1.93 ± 0.14
o__Gaiellales	0.59 ± 0.02	3.56 ± 0.21	2.32 ± 0.05	5.66 ± 0.22
*Gaiella*	0.57 ± 0.01	1.27 ± 0.16	1.07 ± 0.04	3.07 ± 0.27
c__KD4-96	0.57 ± 0.11	1.55 ± 0.20	2.23 ± 0.14	0.38 ± 0.08
*Bacillus*	0.37 ± 0.09	2.01 ± 0.08	1.56 ± 0.19	1.02 ± 0.33
*Burkholderia*	0.03 ± 0.00	0.70 ± 0.10	4.07 ± 0.24	7.19 ± 0.41
*Enterobacter*	0.00 ± 0.00	1.20 ± 0.07	0.10 ± 0.02	3.64 ± 4.39

*. YR, XR, BR, and QR refer to the sampling sites Yinggebao, Xinyang, Baicheng, and Qiubei, respectively. ASmix represents mixed inoculation with the two test strains.

**Table 3:** Physicochemical characteristics in soils from different sampling sites.

Sampling Sites	Soil Trait ^#^					
	OM g/kg	TN g/kg	AP mg/kg	AK mg/kg	pH	TS g/kg
Xinyang	26.40 ± 0.44 a ^$^	1.36 ± 0.02 a	7.47 ± 0.31 a	127.33 ± 2.31 a	7.47 ± 0.15 b	1.53 ± 0.06 bc
Qiubei	23.33 ± 0.70 b	1.06 ± 0.02 b	9.53 ± 0.42 b	226.67 ± 1.15 b	5.87 ± 0.21 a	1.07 ± 0.15 a
Yinggebu	14.6 ± 0.53 d	0.83 ± 0.02 d	7.20 ± 0.70 a	146.33 ± 1.53 c	8.20 ± 0.10 c	1.37 ± 0.06 b
Baicheng	21.9 ± 1.05 c	0.94 ± 0.07 c	10.13 ± 0.23 b	94.00 ± 1.73 d	8.23 ± 0.15 c	1.63 ± 0.06 c

^#^ OM—organic matter, TN—total nitrogen, AP—available phosphate, AK—available potassium, TS—total salts. ^$^ Statistical analysis was performed based on the three repeats of each sampling site. Data are average (*n* = 3) ± standard deviation. Statistical differences between sites are indicated with different letters (*p* < 0.05).

## Data Availability

The raw 16S rRNA gene sequencing reads have been deposited into the NCBI Sequence Read Archive (SRA) database under the accession number of PRJNA1021023.

## Supplementary Materials

The following supporting information can be downloaded at: https://www.mdpi.com/article/10.3390/plants12193421/s1, Table S1: Comparative experimental design of rhizosphere microbial diversity of chickpea. Supplementary Figure S1. The four soil sampling sites from China. Supplementary Figure S2. Results of LEfSe multi-level species differences in rhizosphere soil microbial genera between the new and old areas of chickpea cultivation. Supplementary Figure S3. The correlation between rhizobial composition and soil characteristics from different sampling sites.
