# Supplementary material for: The Effect of Different Rhizobial Symbionts on the Composition and Diversity of Rhizosphere Microorganisms of Chickpea in Different Soils
Source: Plants (Basel). 2023 Sep 28;12(19):3421. doi: 10.3390/plants12193421 (PMC10575130; doi:10.3390/plants12193421)
Supplement: Supplementary file 1 [file plants-12-03421-s001.zip › plants-2603883-supplementary.pdf]

Table S1. Comparative experimental design of rhizosphere microbial diversity of chickpea.

Sampling sites	Processing group	Amount of inoculum (mL)	Sample code
Yinggebao, Xinjiang Province	blank control	1.0 of TY broth	YRASNI
	single inoculation USDA 3378^T	1.0	YRAS3378
	single inoculation CCBAU 83963^T	1.0	YRAS83963
	mixed inoculation	0.5 each	YRASmix
Baicheng, Jilin Province	blank control	1.0 of TY broth	BRASNI
	single inoculation USDA 3378^T	1.0	BRAS3378
	single inoculation CCBAU 83963^T	1.0	BRAS83963
	mixed inoculation	0.5 each	BRASmix
Xinyang, Henan Province	blank control	1.0 of TY broth	XRASNI
	single inoculation USDA 3378^T	1.0	XRAS3378
	single inoculation CCBAU 83963^T	1.0	XRAS83963
	mixed inoculation	0.5 each	XRASmix
Qiubei, Yunnan Province	blank control	1.0 of TY broth	QRASNI
	single inoculation USDA 3378^T	1.0	QRAS3378
	single inoculation CCBAU 83963^T	1.0	QRAS83963
	mixed inoculation	0.5 each	QRASmix

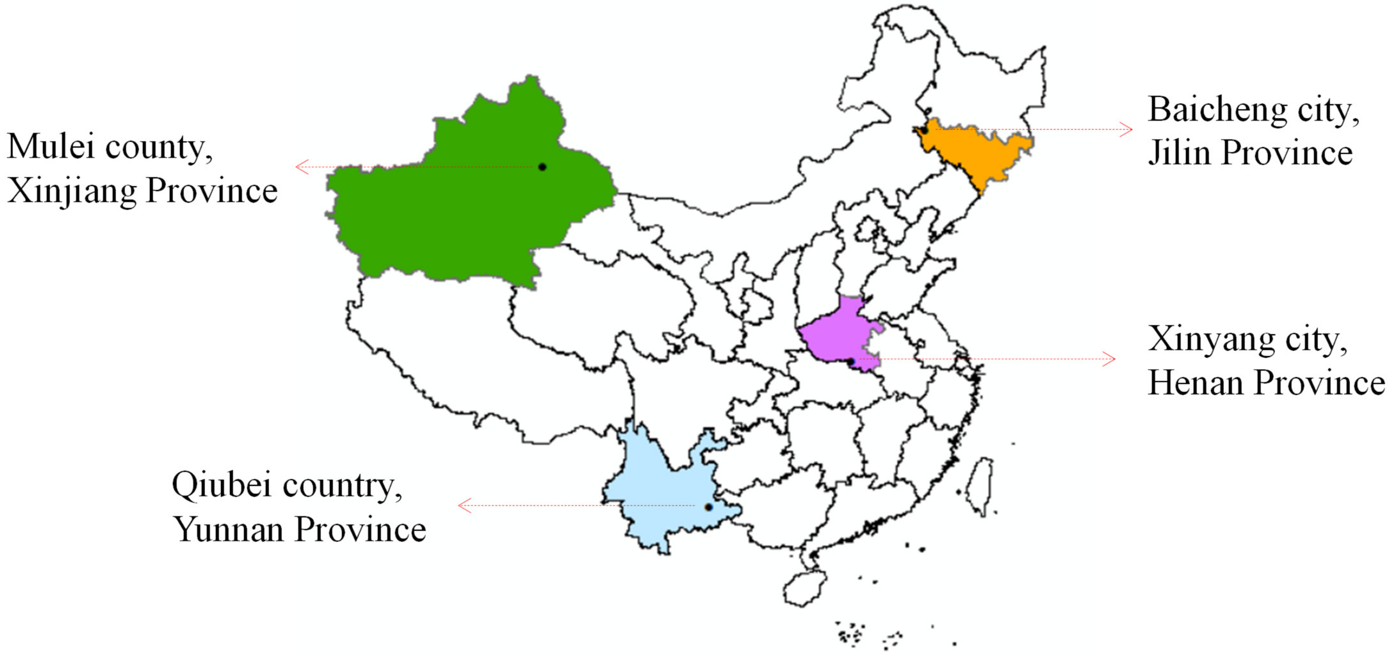

Supplementary Fig. S1. The four soil sampling sites from China.

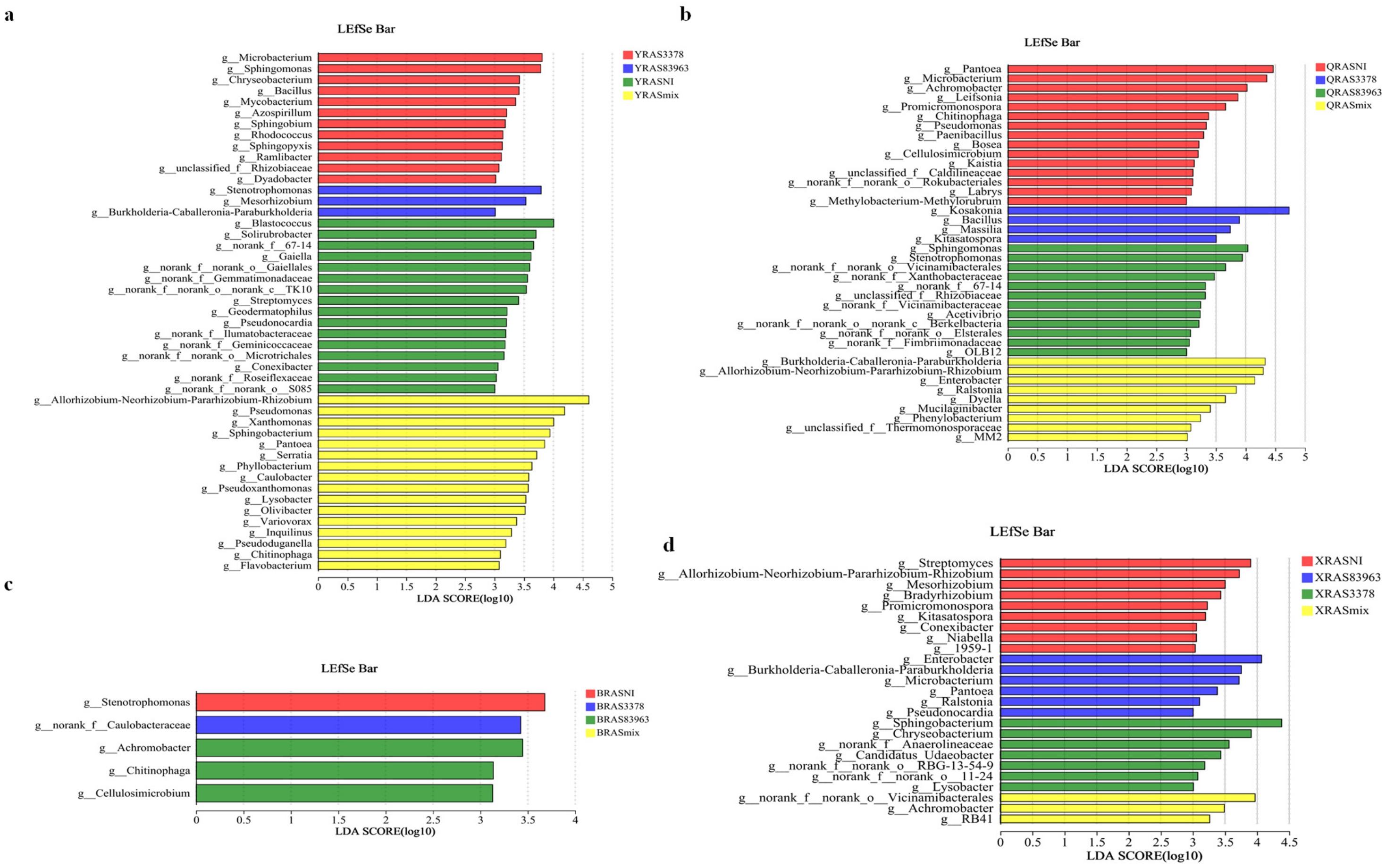

Supplementary Fig. S2. Results of LEfSe multi-level species differences in rhizosphere soil microbial genera between the new and old areas of chickpea cultivation. **a**: Yinggebao of Xinjiang Province. **b**: Qiubei of Yunnan Province. **c**: Baicheng of Jilin Province. **d**: Xinyang of Henan Province. Different colors represent significant relative abundance in rhizosphere soil samples under different treatments. Only microorganisms with LDA score >3.0 are displayed for the increased microorganisms.

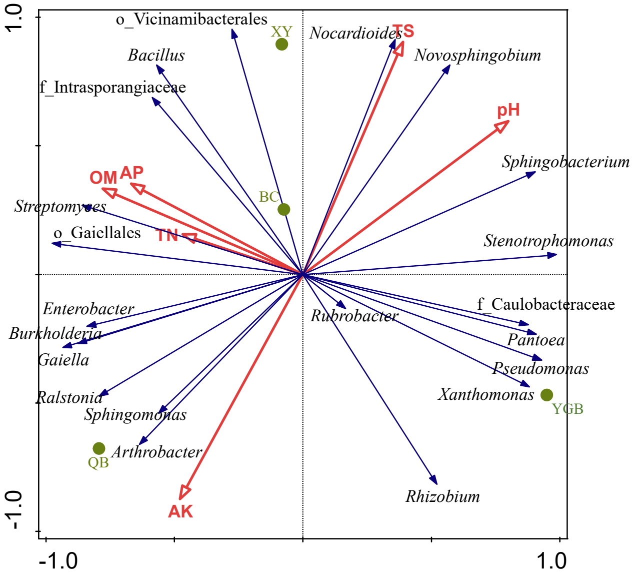

Supplementary Fig. S3. The correlation between rhizobial composition and soil characteristics from different sampling sites. The green dots represent different sampling locations. The red arrows represent soil physicochemical factors. The blue arrows represent the most abundant bacterial genera (> 1% relative abundance) in rhizosphere of chickpea grown in different soil samples inoculated with mixture of *M. ciceri* USDA3378^{T} and *M. muleiense* CCBAU 83963^{T}. The longer the red arrow, the greater the influence of that specific soil property on the distribution of the rhizosphere bacteria; the smaller the angle between the red and blue arrows, the closer the relationship between the soil factor and the rhizosphere bacteria.
